# A Comparison Between Late Preterm and Term Infants on Breastfeeding and Maternal Mental Health

**DOI:** 10.1007/s10995-012-1153-1

**Published:** 2012-10-07

**Authors:** Sheila W. McDonald, Karen M. Benzies, Jenna E. Gallant, Deborah A. McNeil, Siobhan M. Dolan, Suzanne C. Tough

**Affiliations:** 1 Department of Paediatrics, Faculty of Medicine, University of Calgary, Calgary, AB Canada; 2 Faculty of Nursing, University of Calgary, Calgary, AB Canada; 3 Department of Population and Public Health, Alberta Health Services, Calgary, AB Canada; 4 Department of Obstetrics and Gynecology and Women’s Health, Albert Einstein College of Medicine/Montefiore Medical Center, Bronx, NY USA; 5 Department of Community Health Sciences, Faculty of Medicine, University of Calgary, Calgary, AB Canada

**Keywords:** Breastfeeding, Late preterm, Maternal mental health, Postpartum

## Abstract

The objective of this study was to compare breastfeeding, postpartum mental health, and health service utilization between a group of late preterm (LP) maternal infant pairs and term counterparts. Data was drawn from a prospective community-based cohort in Calgary, Alberta. Bivariate and multivariable analyses were performed. LP infants were more likely to have had a longer median length of stay after birth (*P* < 0.001) and a higher re-hospitalization rate at 4-months (*P* < 0.001) compared to term infants. Mothers of LP infants were more likely to report immediate breastfeeding difficulties (*P* < 0.001) and earlier cessation of breastfeeding at 4-months postpartum (*P* = 0.008). Multivariable analyses revealed that LP status was an independent risk factor for excessive symptoms of maternal anxiety (OR = 2.07; 95 % CI = 1.08,3.98), but not for depression, stress, or low parenting morale. LP infants and their families are a vulnerable population with unique developmental trajectories. Further longitudinal research is required.

## Introduction

It has been well established that being born preterm (<37 weeks gestational age) is associated with adverse health and development outcomes for the infant that extend into the early childhood and beyond. These include neonatal morbidities, delays in motor, cognitive, and behavioral functioning during early childhood, and increased risk for chronic disease later in life [1–4]. Preterm births, however, comprise a heterogeneous group with accepted designations of risk comprising very early (<32 weeks), early (32^0/7^–33^6/7^ weeks), and late preterm births (34^0/7^–36^6/7^ weeks). The term, ‘late preterm’ (LP) was introduced by the National Institute of Child Health and Human Development in 2005 to replace the previous descriptor, ‘near term’, to more accurately capture this high risk group as preterm, requiring individual treatment according to their specific needs [5]. In the United States, LP births account for approximately 70 % of all preterm deliveries [6], with Canadian data reporting a similarly high proportion (74 %) [7]. Recent trends have shown an overall increase in the preterm birth rate; especially conspicuous in the United States, the preterm birthrate has increased by 36 % in 25 years, with increases in LP births accounting for much of this rise [8]. Indeed, between 1990 and 2005, US statistics show that while the rate of births before 34 weeks gestation has remained unchanged, the LP birthrate has increased during the same time period [9, 10]. Although a slight decline in the LP birth rate in 2007 in the US has been reported [11], overall, LP births are both the fastest growing and largest subgroup of preterm births in North America [8].

Often comparable in birth weight and appearance to term infants, LP infants lag in development across a number of domains rendering them medically vulnerable [12, 13]. LP newborns are frequently cared for in the general postpartum care units despite their higher risk of physiological issues such as feeding difficulties, weight loss, respiratory distress, hypoglycemia, hypothermia, hyperbilirubinemia, postural instability, immature oro-motor development, and lethargy compared to term infants [13–18]. Many of these physiological dangers place LP infants at increased risk of morbidity and mortality compared to their term counterparts [19–21]. These LP infants often require additional observation time in the hospital, continued therapies, and are more likely to be re-admitted, or have a longer initial length of stay post delivery [21, 22], all of which translate into increased demands on the healthcare system, as well as to mothers, families, and caregivers.

While the extant literature in this area suggests that short-term medical risks are common for LP infants, more research is needed to examine long-term outcomes [23]. There are only a limited number of reports documenting later-life outcomes for LP infants, and more prospectively collected data is required to validate existing observations [24]. One recent prospective investigation, drawing from a longitudinal study of cognitive and behavioral outcomes associated with low birth weight, found that at 6 years of age, children that were born LP were more likely to have lower IQ and behavior problems compared to term counterparts even after controlling for maternal IQ and socioeconomic status [25]. These findings align with an earlier population-based study which found increased risk for adverse school-age outcomes for LP infants [26, 27]. Although null findings have also been reported [28], a recent systematic review on early childhood development of LP infants concluded that LP infants are at increased risk of poor developmental outcomes (e.g., neurodevelopmental disabilities, poor educational ability, early-intervention requirements) up to school age compared to term infants [23].

Potential mediators and moderators of threats to development are also understudied for children born LP; these outcomes include maternal and family health factors [2] and emotional responses. Some researchers have suggested that mothers of LP infants are at risk of persistent anxiety and depression [29]. This is of particular concern given the influence of maternal mental health after birth on infant and child development [30–34]. Reports on maternal symptomatology associated with *early* preterm births have noted increased rates of depression, anxiety, and stress in both the short and long-term [35–39]. Of the few studies that have examined maternal mental health with respect to the LP infant, similar findings have been reported [29, 40], yet given differences in study design, timing of assessment, control for confounding variables, and source of sample population, more research is needed to inform the evidence base that drives clinical practice and policy recommendations. One small study that examined maternal mental health on day 3–4 postpartum found increased levels of anxiety, depression, and stress among mothers of LP infants compared to term infants, which, in turn, led to earlier cessation of breastfeeding [41]. A recent study examined emotional distress among mothers of LP infants up to 1 month postpartum and found increased levels of anxiety, depression, post-traumatic stress, and worry compared to mothers of term infants [40], yet limitations noted by the authors such as small sample size, lack of baseline emotional distress, and high-risk referral source, indicate that further research is required.

In summary, LP births constitute a growing public health concern yet knowledge gaps remain concerning long-term outcomes for infants and their families. In particular, postpartum mental health constitutes a significant area of investment for further research to identify effective prevention and intervention strategies to support these families. This study evaluated the independent influence of LP birth on maternal symptoms of anxiety, depression, and stress, while controlling for maternal demographic factors and mental health history. Furthermore, this study examined parenting morale among LP mothers, a measure of psychological well-being that taps into a mother’s internal coping resources. Finally, this study compared immediate postnatal experiences including breastfeeding experiences and health care utilization between a group of LP maternal-infant pairs and their term counterparts.

## Materials and Methods

The All Our Babies (AOB; n = 1,654) study is a community-based prospective pregnancy cohort study in Calgary, Alberta that began in 2008. The overall aims were to examine maternal well-being during the perinatal period and infant outcomes such as preterm birth, and to identify the current barriers and facilitators to accessing prenatal care. Participation in the study involved the completion of three mailed questionnaires (<24 weeks, 34–36 weeks, 4-months postpartum) and consent for linkage to medical records. The response rate across the three time points was over 80 %, with pregnancy loss or lost to follow-up constituting the most common reasons for attrition.

The questionnaires, which included standardized scales and investigator derived questions, inquired about socio-demographics, lifestyle, heath care utilization, stress, depression, anxiety, birth outcomes, parenting morale, and breastfeeding, among other variables. Data were cleaned to align with pre-existing data coding and cleaning guidelines [42, 43]. For the present study, data were analyzed using the SPSS statistical software program (Version 19.0).

For the purposes of the present study, we restricted the sample (n = 1,227) to women giving birth to a singleton infant and excluded those with a self-reported gestational age of 37 weeks in order to limit the potential for misclassification of term status. Two groups of women were compared: a group of mothers who self-reported a gestational age at delivery between 34 and 36 weeks (late-preterm; n = 77) and those who delivered at 38 weeks or more (n = 1,150). For the present analysis, the following validated instruments were used: the Edinburgh Postnatal Depression Scale (EPDS) [44], the Cohen Perceived Stress Scale (PSS) [45], the Spielberger State Anxiety Index (SSAI) [46], and the Parenting Morale Index [47], to measure maternal symptoms of depression, stress, anxiety, and parenting morale, respectively. Example items for each standardized scale and other variables of interest for the present study are shown in section Appendix.

LP and term groups were compared on maternal characteristics as well as prenatal and immediate postpartum experiences, using Chi Square test/Fisher’s exact test for categorical variables and Independent Samples *t* test/Mann–Whitney U for continuous variables. Distributional assumptions were checked prior to analysis to ensure test appropriateness (parametric vs. non-parametric). Multivariable logistic regression models were performed to examine the extent to which birth status was a significant independent predictor of excessive symptoms of anxiety, depression, stress, and parenting morale at 4-months postpartum, controlling for known risk factors and confounding variables. Demographics, prepregnancy maternal characteristics, and select pregnancy variables that were significant at the bivariate level (*P* < 0.1) were considered for inclusion in the multivariable analyses as confounding variables. Excluded predictors or variables not significant at the bivariate level were added to the final models, one at a time, to ensure that important confounding variables were not missed. Factors that were considered to be intermediate variables on the causal pathway between birth status and postpartum mental health outcomes (e.g., breastfeeding experiences, health care utilization) were not included in multivariable models as confounding variables as their inclusion could lead to bias of the association between birth status and mental health outcomes (i.e., ‘overadjustment’). Blocks of variables were manually entered in a hierarchal fashion: demographic variables were entered first, followed by prepregnancy characteristics, prenatal mental health variables, and, finally, birth status. Finally, goodness-of-fit statistics and regression diagnostics were performed [48].

This study was approved by the Conjoint Health Research Ethics Board of the University of Calgary. Participants provided informed consent at the time of recruitment and were provided copies for their records.

## Results

Comparison of birth status indicated that mothers of LP infants were significantly more likely to be non-Caucasian (37.3 vs. 22.2 %; *P* = 0.003), to report lower household income levels (27.4 vs. 16.9 %; *P* = 0.02), and to have had a previous preterm birth (12.0 vs. 9.1 %; *P* = 0.01) (Table 1). There were no significant differences between the two groups in terms of the other demographic and prepregnancy maternal characteristics examined. Table 2 shows comparisons between the two groups on pregnancy variables, delivery and health care utilization variables, and breastfeeding outcomes. Significant differences between the two groups were found for all factors that were compared except for substance use during pregnancy and delivery method. As expected, LP singleton infants were significantly more likely than term singleton infants to be in the low birth weight category (<2,500 g) (29.9 vs. 1.0 %; *P* < 0.001), and to have experienced a longer median length of stay in the hospital after birth (4 vs. 1.5 days; *P* < 0.001). Additionally, mothers of LP infants were more likely to have reported excessive prenatal anxiety (40.0 vs. 27.0 %; *P* = 0.015) and prenatal depression symptoms (19.5 vs. 10.0 %; *P* = 0.009), and to have experienced breastfeeding difficulties. In terms of the latter, LP infants were less likely to have breastfed within 24 h after birth (78.7 vs. 97.5 %; *P* < 0.001) as well as before leaving the hospital (78.1 vs. 90.8 %; *P* < 0.001), and to be breastfeeding at 4 months (69.3 vs. 81.7 %; *P* = 0.008). Mothers of LP infants were more likely to report being unsuccessful on their first attempt at breastfeeding (50.7 vs. 75.0 %; *P* < 0.001) and to have consulted with a lactation professional before leaving the hospital (73.0 vs. 39.6 %; *P* < 0.001). Finally, LP infants were significantly more likely than term infants to be re-hospitalized before 4 months (14.5 vs. 3.8 %; *P* < 0.001).

**Table 1:** Comparison of demographic and maternal characteristics (prepregnancy) between the late-preterm and full-term groups

Characteristic	Total sample (n = 1,227) n (%)	LPs (n = 77) n (%)^a^	Terms (n = 1,150) n (%)^a^	*P* value
*Maternal age*				
<25 years	75 (6.3)	8 (11.0)	67 (6.0)	0.24
25–34 years	871 (73.3)	51 (69.9)	820 (73.5)	
35+ years	243 (20.4)	14 (19.2)	229 (20.5)	
*Education*				
High school or less	123 (10.1)	7 (9.3)	116 (10.1)	0.72
College, trade, university	893 (73.3)	53 (70.7)	840 (73.4)	
Grad school	203 (16.7)	15 (20.0)	188 (16.4)	
*Household income*				
<60,000	207 (17.5)	20 (27.4)	187 (16.9)	0.02
60,000+	974 (82.5)	53 (72.6)	921 (83.1)	
*Marital status*				
Married/common-law	1,156 (94.9)	73 (97.3)	1,083 (94.8)	0.58
Other	62 (5.1)	2 (2.7)	60 (5.2)	
*Ethnic origin*				
White/Caucasian	937 (76.9)	47 (62.7)	890 (77.8)	0.003
Other	282 (23.1)	28 (37.3)	254 (22.2)	
*Born in Canada*				
Yes	942 (77.2)	53 (70.7)	889 (77.6)	0.16
No	278 (22.8)	22 (29.3)	256 (22.4)	
*History of depression*				
No	841 (68.9)	56 (74.7)	785 (68.7)	0.27
Yes	379 (31.1)	19 (25.3)	360 (31.4)	
*History of other mental health problems*				
No	1,128 (91.9)	69 (92.0)	1,059 (92.7)	0.84
Yes	90 (7.4)	6 (8.0)	84 (7.3)	
*History of abuse*				
No	851 (71.7)	47 (67.1)	804 (72.0)	0.38
Yes	336 (28.3)	23 (32.9)	313 (28.0)	
*Parity*				
0	626 (51.6)	43 (57.3)	583 (51.2)	0.30
1+	588 (48.4)	32 (42.7)	556 (48.8)	
*Previous preterm birth*				
No	1,155 (94.7)	66 (88.0)	1,089 (95.2)	0.01
Yes	64 (15.3)	9 (12.0)	55 (9.1)	
*Previous lbw birth*				
No	1,183 (97.0)	29 (85.3)	578 (94.9)	0.07
Yes	36 (3.0)	5 (14.7)	31 (5.1)	
*Used active method to get pregnant*				
No	1,151 (94.4)	74 (98.7)	1,077 (94.1)	0.12
Yes	68 (5.6)	1 (1.3)	67 (5.9)	

*lbw* low birth weight

^a^Denominator varies due to missing values for some variables

**Table 2:** Comparison of pregnancy, delivery, and breastfeeding experiences between the late-preterm and full-term groups

Characteristic	LPs (n = 77) n (%)/median (IQR)^a^	Terms (n = 1,150) n (%)/median (IQR)^a^	*P* value
*Pregnancy*			
Smoked during pregnancy	8 (11.0)	98 (8.7)	0.50
Consumed alcohol during pregnancy	27 (37.0)	454 (40.0)	0.61
Prenatal depression	15 (19.5)	115 (10.0)	0.009
Prenatal anxiety	30 (40.0)	309 (27.0)	0.015
*Delivery and health care utilization*			
C-section delivery	19 (24.7)	243 (21.2)	0.47
Birth weight <2,500 g	23 (29.9)	11 (1.0)	<0.001
Length of infant hospital stay (days)	4.00 (8.24)	1.50 (1.00)	<0.001
Infant rehospitalization rate between discharge and 4-months	11 (14.5)	42 (3.8)	<0.001
*Breastfeeding*			
Breastfeeding within 24 h after delivery	59 (78.7)	1,096 (97.5)	<0.001
Successful breastfeeding on first attempt	38 (50.7)	841 (75.0)	<0.001
Breastfeeding before leaving hospital	57 (78.1)	957 (90.8)	<0.001
Saw a lactation consultant before leaving hospital	54 (73.0)	434 (39.6)	<0.001
Still breastfeeding at 4-months postpartum	52 (69.3)	919 (81.7)	0.008

^a^Denominator varies due to missing values for some variables

We performed four separate multivariable logistic regression models for each maternal mental health outcome at 4 months postpartum. We categorized mothers as reporting either excessive symptoms or not, according to validated or accepted cut-offs as per the literature (40 and above for anxiety) [46] and 13 and above for depression [44]. For perceived stress symptoms and parenting morale at 4-months postpartum, we considered scoring above the 80th percentile (stress) or below the 20th percentile (parenting morale) of the distribution as manifestation of excessive stress and low parenting morale, respectively. Each multivariable model controlled for confounding variables, a number which are also established risk factors for poor postpartum mental health (e.g., history of depression, prenatal depression and prenatal anxiety) [49–51]. Final models are shown in Table 3; each model presents the odds ratios and 95 % confidence intervals for term status as well as those covariates that remained significant at *P* < 0.05. Excluded predictors were added, one at a time, back into the final models to ensure that important confounding variables were not missed in the presence of other variables in the model. The final multivariable logistic regression analysis for anxiety symptoms at 4-months postpartum showed that mothers of LP infants were significantly more likely than mothers of term infants to report excessive anxiety (OR = 2.07, 95 % CI = 1.08,3.98), even after controlling for prenatal anxiety and depression, a history of depression, and household income (Table 3). LP status was not an independent risk factor in multivariable logistic regression models for depression, stress, or parenting morale at 4-months postpartum (Table 3).

**Table 3:** Final multivariable logistic regression models examining the independent contribution of birth status to parenting morale, maternal anxiety, depression, and stress symptomatology at 4-months postpartum

Independent variable	Anxiety OR (95 % CI)	Depression OR (95 % CI)	Stress OR (95 % CI)	Parenting morale OR (95 % CI)
Household income (<$60,000)	1.55 (1.00,2.42)		1.60 (1.06,2.42)	
Nulliparous				0.58 (0.42,0.80)
History of depression	2.90 (1.99,4.24)	2.79 (1.55,5.03)	2.24 (1.57, 3.20)	2.36 (1.69,3.30)
Prenatal anxiety	5.16 (3.44,7.74)	3.95 (2.01,7.78)	3.88 (2.66,5.66)	3.54 (2.49,5.03)
Prenatal depression	2.64 (1.64,4.27)	3.60 (1.92,6.75)	3.13 (1.97,4.97)	1.82 (1.15,2.88)
Ever experienced abuse				1.50 (1.07,2.10)
LP birth status	**2.07 (1.08,3.98)**	0.73 (0.24,2.23)	1.34 (0.69,2.61	1.23 (0.65,2.35)

Only those variable that remained significant at *P* < 0.05 in the final multivariable model for each respective outcome are shown

Significance value for bolded OR is *P* = 0.029

## Comment

Using data from a community-based prospective pregnancy cohort in Calgary, Alberta, we compared maternal characteristics, postnatal experiences, and symptoms of maternal anxiety, depression, stress, and parenting morale at 4-months postpartum between a group of mothers of LP infants (n = 77) and a group of mothers of term infants (n = 1,150). The demographic profile of the AOB sample, in general, is representative of the urban pregnant and parenting population in Canada [52]. Controlling for baseline measures of distress, LP birth was associated with excessive anxiety symptoms at 4-months postpartum but not with depressive symptoms, stress, or with parenting morale. In addition, immediate postnatal healthcare utilization and breastfeeding outcomes were significantly different between the two groups.

The results corroborate previous studies that suggested that LP infants and their mothers are at risk of a number of poor outcomes both immediately after delivery and the months following birth. A recent review article reported increased hospitalization rates and delayed hospital discharge due to ‘poor feeding’ as compared to term infants or non-breastfeeding LP infants [53]. In our study we found similar results in terms of increased hospitalization rates and longer hospital stay after birth for LP infants compared to term births, findings consistent with previous reports for LP infants [21, 22, 54]. Our study did not allow for examination of specific reasons for delayed discharge or re-hospitalization, yet given their low breastfeeding initiation and success rates, poor feeding could be a contributing factor. There is some evidence to suggest earlier breastfeeding cessation rates for LPs compared to terms [53]. At 4 months postpartum, 69 % of the LP group was still breastfeeding compared to 82 % of the term group. Our lower rates of breastfeeding initiation and success as reported by mothers of LP infants are in line with reports that LP infants present challenges for breastfeeding success and duration due to inadequate milk transfer [55], physiological issues in the infant (e.g., cardio respiratory insufficiency, metabolic disturbances, uncoordinated suck-swallow-breathe mechanism), and NICU admission and consequent maternal-infant separation [5, 17, 53, 56]. Given the longitudinal design of the AOB study, planned analyses include examining trajectories of feeding experiences across the early life course among LPs. Longitudinal data will help to further understand feeding trajectories, including exclusive breastfeeding rates according to the World Health Organization criteria (until 6 months of age and continuing for up to 2 years) [57].

Maternal anxiety and depression are often co-morbid conditions that occur in the postpartum [58–60]. Given the limited number of investigations on maternal mental health outcomes for mothers of LP infants, our community-based study is a welcome addition to supplement the current evidence base. The multivariable regression results of our study identified increased anxiety compared to other symptoms of poor mental health at 4-months post-delivery for mothers of LP infants, which is in contrast to a recent report of emotional responses of mothers of LP and term infants [40]. The authors of the latter found that mothers of LP infants had greater emotional distress on standardized measures for anxiety, depression, posttraumatic stress, and worry immediately following delivery and at one-month postpartum. As our follow-up time frame extended to 4-months postpartum, it is possible that initial levels of depressive and stress symptoms decrease over time. Yet this suggestion is in contrast to another recent study that reported elevated levels of both anxiety and depression 6-months after delivery among mothers of LP infants compared to term infants [29]. Indeed, the interplay between stress, anxiety, depression and parenting morale as well as the specificity for long-term anxiety beyond 4-months is an area for further investigation.

Our study, in contrast to the few previous studies examining postpartum mental health among mothers of LP infants, had the advantage of having collected information on, and therefore controlling for, a history of mental health difficulties and prenatal anxiety and depression in multivariable models for postpartum distress. It could be that significant associations between LP birth status and postpartum maternal depression and posttraumatic stress (for example) previously reported were due to residual confounding, as this type of information was unavailable in those studies. Additional strengths of the present study include its prospective design and population-based sample. By comparing a LP group to a term group with a lower bound for gestational age at 38 weeks versus the standard 37 weeks, we minimized misclassification of group status.

Given that all factors measured in the current study were based on maternal self-report, some error may have been introduced. We dichotomized mental health symptomatology as high or low; although previous studies have examined symptoms in continuous form, they also present categorical findings according to clinical cut-offs [40] and in the top quartile of the distribution [29]. The continuous scores showed evidence of skewness in our sample; for this reason and given the presence of established cut-offs in the literature for most of the scales, we used multivariable logistic regression to build the final models. Of note, a similar pattern of results was seen when linear regression modeling was done using transformed scores (results not shown). Regardless of how these scores are ultimately handled in analyses, all studies to date share the limitation imposed by the self-report nature of maternal mental health symptoms and therefore conclusions on ‘caseness’ or diagnostic classification cannot be made. However, the prospective nature of data collection in our study minimizes the risk of recall bias of mental health at each data collection time point. An avenue for further research would be to examine the gradient of gestational age with maternal mental health symptomatology. The recent systematic review by McGowan et al. [23] on early childhood outcomes of LP infants concluded that LP infants fall between very preterm infants and term infants on a developmental outcome continuum, suggesting a ‘scale’ of prematurity, and an inverse, linear association between gestational age and risk of longer-term disability. Similarly, results from a recent population based cohort study [61] suggest a dose response effect of prematurity on health outcomes at 3 and 5 years of age. Such gradient effects warrant further investigation.

Other possible reasons for discrepant results for postpartum mental health between our study and previous ones may concern the demographic and socioeconomic profile of study participants, as our study tended to attract women with higher than average education and household incomes. Given differences in the demographic profile of our participants, compared to the target populations of the relatively few studies on postpartum mental health among LP mothers [29, 40], which tend to be more diverse and/or characterized by low socioeconomic status, this data suggest that trajectories of maternal mental health among LP mothers of relatively affluent socioeconomic status may also differ.

In summary, results from our study suggest that LP infants are a vulnerable group, impacting both infant and maternal mental health, in particular, immediate breastfeeding challenges and excessive anxiety at 4-months postpartum. Whether this vulnerability extends across time remains largely unexplored. Given the unique challenges of LP birth [13], it is imperative that further research into the precursors, correlates, and outcomes associated with a LP birth continue in order to best support their specific maternal and infant needs.

## Appendix

See Table 4.

**Table 4:** Select variables assessed in the AOB study used in the present study

Candidate variable (data collection time point)^a^	Source/phrasing	Scoring and/or coding information
*Standardized Scales*		
Prenatal and postnatal depressive symptoms (t1–t3):	Edinburgh Postnatal Depression Scale	10 item questionnaire. Each item rated on a 4-point Likert scale from 0 to 3. After reverse scoring for some items, a total score is derived (range 0–30). Higher scores reflect increased depression. We used a standard cut-off as per the literature (13 and above) to identify high depressive symptoms. Participants are instructed to respond to each item with respect to the past 7 days. Example items include: “In the past 7 days, I have felt sad or miserable”; “In the past 7 days, I have been so unhappy that I have been crying”
Prenatal and postnatal stress symptoms (t1–t3):	Perceived Stress Scale	10 item questionnaire. Each item rated on a 5-point Likert scale from 0 to 4. After reverse scoring for some items, a total score is derived (range 0–40). Higher scores reflect increased stress. A cut-off at the 80th percentile of the sample distribution was used to classify women as manifesting high stress symptoms. Participants are instructed to respond to each item with respect to the past month. Example items include: “Felt unable to cope with all the things you had to do”; “Felt that difficulties were piling up so high that you couldn’t overcome them”
Prenatal and postnatal anxiety symptoms (t1–t3):	Spielberger State Anxiety Inventory	20 item questionnaire. Each item rated on a 4-point Likert scale from 1 to 4. After reverse scoring for some items, a total score is derived (range 20–80). Higher scores reflect increased anxiety. We used an established cut-off of 40 or more to classify women as experiencing high anxiety symptoms. Participants are instructed to respond to each item as of right now (in this moment). Example items include: “I feel anxious”; “I feel worried”; “I feel nervous”
Parenting morale symptoms (t3):	Parenting Morale Index	10 item questionnaire. Each item rated on a 5-point Likert scale from 1 to 5. After reverse scoring for some items, a total score is derived (range 10–50). Higher scores reflect better parenting morale. A cut-off at the 20th percentile of the sample distribution was used to classify women as reporting low parenting morale. Participants are instructed to respond to each item with respect to their daily life as a parent. Example items include: “optimistic”; “contented”; satisfied”
*Select single-item questions*		
Parity (t1):	Combination of the following: “Have you been pregnant before?”; “Have you ever experienced a miscarriage, stillbirth, abortion, neonatal death or live birth?”	Yes/no
History of depression (t1):	“Have you ever experienced feeling sad, blue, depressed or down for most of the time for at least 2 weeks?”	Yes/no
Ever experienced abuse (t2):	Based on 5 questions: “Have you ever experienced physical abuse; emotional abuse; sexual abuse; financial abuse; or neglect?”	Yes/No
Term/birth status (t3):	“How many weeks pregnant were you when your baby was born?”	Number of weeks. We categorized gestational age at birth into two groups: late preterms (34–36^6/7 ^weeks gestational age at birth) and terms (38+ weeks gestational age at birth)
Length of infant hospital stay (days; t3):	“After your baby was born, how long was their hospital stay?”	Number of days
Infant rehospitalization rate between discharge and 4-months (t3):	“Has your baby stayed overnight in the hospital (not including when he/she was first born nor NICU)? How many times each?”	Selected/non selected; number of times. This variable was dichotomized into none versus at least once
Breastfeeding within 24 h after delivery (t3):	“Was your first attempt at breastfeeding your baby within 24 h of giving birth?”	Yes/no
Successful breastfeeding on first attempt (t3):	“Were you able to successfully breastfeed on your first attempt?”	Yes/no
Breastfeeding before leaving the hospital (t3):	“Were you able to breastfeed before you went home from the hospital?”	Yes/no
Saw a lactation consultant before leaving hospital (t3):	“Did you see a lactation consultant before you went home from the hospital?”	Yes/no
Still breastfeeding at 4-months postpartum (t3):	“Are you still breastfeeding your baby?”	Yes/no

^a^ *t1* <24 weeks gestational age, *t2* 34–36 weeks gestational age, *t3* 4-months postpartum
